# Developmental Enamel Defects in Children from the Southern Region of Ecuador

**DOI:** 10.3390/children9111755

**Published:** 2022-11-16

**Authors:** Eleonor Vélez-León, Alberto Albaladejo-Martínez, Edisson-Mauricio Pacheco-Quito, Ana Armas-Vega, Andrés Delgado-Gaete, Doménica Pesántez-Ochoa, María Melo

**Affiliations:** 1 Department of Surgery, Faculty of Medicine, University of Salamanca, 37007 Salamanca, Spain; 2 Academic Unit of Health and Wellness, Faculty of Dentistry, Catholic University of Cuenca, Cuenca 010105, Ecuador; 3 Innovation and Pharmaceutical Development in Dentistry Research Group, Faculty of Dentistry, Head of Research and Innovation, Catholic University of Cuenca, Cuenca 010105, Ecuador; 4 School of Dentistry, Hemisferios University, Quito 170527, Ecuador; 5 Ministry of Public Health, Cuenca 010105, Ecuador; 6 Department of Stomatology, Faculty of Medicine and Dentistry, University of Valencia, 46010 Valencia, Spain

**Keywords:** dental enamel, permanent dentition, prevalence, dental enamel hypoplasia

## Abstract

Developmental defects of enamel (DDEs) are widely observed in children and are related to the appearance of dental caries, malocclusion, tooth sensitivity, and unfavorable esthetic conditions. The objective of this cross-sectional study was to determine the prevalence and distribution of enamel defects present in children aged 6 to 12 years in the provinces located in southern Ecuador. A total of 1606 schoolchildren were examined under the World Health Organization criteria for diagnosis of DDEs. The results are presented using percentage frequency measures and chi-square associations. Some types of DDEs were presented by 50% of the schoolchildren, mainly diffuse opacity, with no statistical differences according to place of residence and/or environment, sex, and age (*p* > 0.05). In Ecuador, it is necessary to carry out studies on the factors that trigger enamel defects, since they may be associated with the high prevalence of caries already reported in other studies in the country.

## 1. Introduction

Developmental defects of enamel (DDEs) are alterations in the quality and quantity of enamel caused by disruption or damage of the dental organ during the process of amelogenesis [1,2,3]. The permanent dentition begins its mineralization in the 28th week of gestation and is completed at 3 years of life. Any alteration in amelogenesis will result in a quantitative or qualitative defect whose presentation will depend on the stage of development in which the lesion occurs, as well as its extension and duration [4,5,6,7]. 

The factors that can interfere with the metabolic process of enamel formation and produce DDEs are generally divided into two groups: acquired and hereditary [1]. The risk factors for acquired DDEs are divided into prenatal, natal, and postnatal factors. Regarding prenatal factors, research has shown a higher frequency of DDEs in children who have suffered intrauterine malnutrition, inadequate nutrition during fetal development, medical complications during pregnancy related to vitamin D deficiency, low calcemic, gestational diabetes, maternal psychological stress, anemia related to hypotension, and frequent exposure to radiographs in the last trimester of pregnancy [5,8,9,10,11]. Regarding natal factors, the literature mentions that the development of DDEs would be influenced by delivery complications, such as premature delivery, low birth weight, vitamin D deficiencies, low calcemic, and low vitamin A levels [1,12]. On the other hand, postnatal risk factors include severe illness in the first 3 years of life, vitamin D deficiency, infectious episodes, thyroid dysfunction, use of drugs such as antiretrovirals, and antibiotics, especially penicillin [13,14]. Several studies have highlighted nutritional status as a risk factor associated with DDEs. Dietary deficiencies during pregnancy and at birth influence the formation of dental enamel [15,16].

Disorders that occur during the early stages of enamel development will result in a reduction in the amount or thickness of enamel, i.e., enamel hypoplasia, which is defined as a deficiency in the amount of enamel resulting from developmental changes. This clinically may present as pits, cracks, or loss of large areas of enamel [1,17]. Conversely, disorders that occur during the calcification and maturation phase of enamel development may result in mineralization deficiencies (hypocalcification) and usually manifest as changes in enamel translucency or opacities that may be diffuse or demarcated [5,18]. Therefore, enamel defects can be studied as a marker of many adverse biological events that occur during the time of their development, and this may have applications in clinical, epidemiological, and anthropological research [5].

In clinical practice, it is of great importance to pay attention to the presence of DDEs, as it may cause esthetic problems, such as staining and morphological alterations [11]. Children with DDEs may experience feelings of anxiety and social embarrassment regarding their dental appearance [5,19]. In addition, in many affected children, there is increased tooth sensitivity due to hypomineralization of enamel and exposed dentin [2,11,20,21,22].

Epidemiological information on DDEs is substantial within and between populations, as it can contribute to the assessment and monitoring of environmental or systemic factors and to the detection of possible etiological factors responsible for their occurrence [23]. In Ecuador, there are no studies of DDEs, but there are data on dental fluorosis in different regions of the country, specifically in the north of Ecuador, where the levels of dental fluorosis are high [24,25]. Since dental enamel is a biological marker of the expression of different types of lesions, we believe it is essential to develop the first epidemiological report of DDEs in the country to determine the prevalence of enamel alterations in schoolchildren aged 6 to 12 years in urban and rural environments of three provinces of Ecuador that make up the southern region of the country.

## 2. Materials and Methods

### 2.1. Design

An observational, quantitative, descriptive, and cross-sectional study was carried out to identify the prevalence of DDEs in school-age children living in urban and rural settings in three provinces located in the southern region of the country. The Board of Directors of the Academic Unit of Dentistry granted approval for this study (Resolution No. 048 CD-2019, approved 14 February 2019). Prior to the study, the parents of the participants were informed and authorized in writing their participation with the delivery of the informed consent.

### 2.2. Sample

For 2019, the estimate of the school population aged 6 to 12 years in the study area was 183,081 schoolchildren. The sample size was calculated by convenience using the EPIDAT 4.0, resulting in 1938 participants. The reliability of calculation considered was 99% (Z = 2.58) with an error of 2.5%.

The study participants had to meet the inclusion criteria, have informed consent, reside in the study areas, not have fixed appliances in the oral cavity that would impede the examination, and be in the mixed or permanent dentition stage. Schoolchildren who presented legal or physical impediments, who did not meet the required age, and with only primary dentition were not included.

After the application of the inclusion and exclusion criteria, the final sample consisted of 1606 participants.

### 2.3. Calibration

The calibration process of the examiners was carried out by certified professionals in the field. The diagnostic criteria were those stipulated by the World Health Organization (WHO) [26,27]. The data collection instrument consisted of forms with the following parameters: identification (first and last names, age, sex), number of the tooth to be examined, tooth surface (vestibular, lingual or palatal and occlusal), type of enamel development defect, and place and environment of residence.

Calibration was performed in several stages under the training of experts in the field of DDEs. The process consisted of a first phase where theoretical training with photographs on the subject was carried out, followed by clinical practice on extracted teeth prepared for the practice. The next phase consisted of clinical examination of two groups of schoolchildren from a school that was not included in the study. Each examiner reviewed the two groups of children accompanied by an assistant, who helped them to record the information on the forms. The calibrated examiners obtained interexaminer Kappa values of 0.8 and intraexaminer values of 0.9.

### 2.4. Diagnostic Criteria

The diagnostic criteria developed were those recommended by the WHO [14], taking into account the following recommendations: (a) In the absence of suspicion of the presence of DDEs, the tooth surface was classified as “Normal” (Key O). (b) Tooth surfaces showing only one DDE not exceeding 1 mm in diameter were classified as “O”. (c) DDEs that could not be easily classified into one of the three basic types were included in “Other defects”. (d) A tooth was considered to be present if any part of the tooth penetrated the mucosa; the alteration found on the visible part was recorded. (e) If more than two-thirds of the surface of a tooth had restorations or extensive caries processes or fractures, they were not examined.

### 2.5. Examination

The institution designated the places for the examination, taking into account that they had natural lighting and ventilation, that the participants had informed consent, and that they did not have fixed intraoral appliances. Prior to the examination, the students brushed their teeth under the supervision of dental students. The examinations were performed by a professional calibrated with a mouth mirror, a periodontal probe, and an artificial light source. The indicator teeth were examined wet to detect DDEs.

### 2.6. Statistical Analysis

The analysis starts with the general analysis of prevalence and then shows the levels of alteration. The prevalence was calculated taking into account that the participant has at least one DDE in the dentition. The prevalence of the types of DDE disorders is presented according to the teeth analyzed by province and age of the participants. The results are expressed as percentage frequency measures. The chi-square statistic was used to establish the association between variables. The IBM^®^ SPSS v.27 (New York, NY, USA) and JASP^®^ 0.16.2 (Amsterdam, The Netherlands) statistical programs were used. The analysis was performed in the SPSS V27 statistical program, and the significance level was 5% (*p* < 0.05).

## 3. Results

A total of 1606 participants from urban and rural settings in three provinces in the south of the country were examined. The prevalence of DDEs was determined by including all individuals who had at least one tooth affected by the disease. 

The prevalence of DDEs according to sex, age, province, and environment was 50%, with no significant differences (*p* < 0.05) when comparing each group. However, the age with the highest prevalence was 8 years (53.1%), and according to sex, females reported a slightly higher rate compared to males (Table 1).

The distribution of the types of DDEs according to sex shows homogeneous values. Diffuse opacity is the most prevalent at all ages. Delimited opacity presents values between 9 and 15% at all years. Finally, hypoplasia presents higher values at 12 years of age (Table 2).

The degree of diffuse opacity registers similar values in all provinces with percentages around 20 to 25%. The same happens with delimited opacity with values around 9%. Regarding hypoplasia, the highest percentage is found in the province of Cañar, showing a higher prevalence in the rural area, and finally, delimited opacity and hypoplasia show lower percentages, even values of 0 in some urban areas (Table 3). Comparison according to the participants’ environment revealed no significant differences (*p* > 0.05).

**Figure 1:**
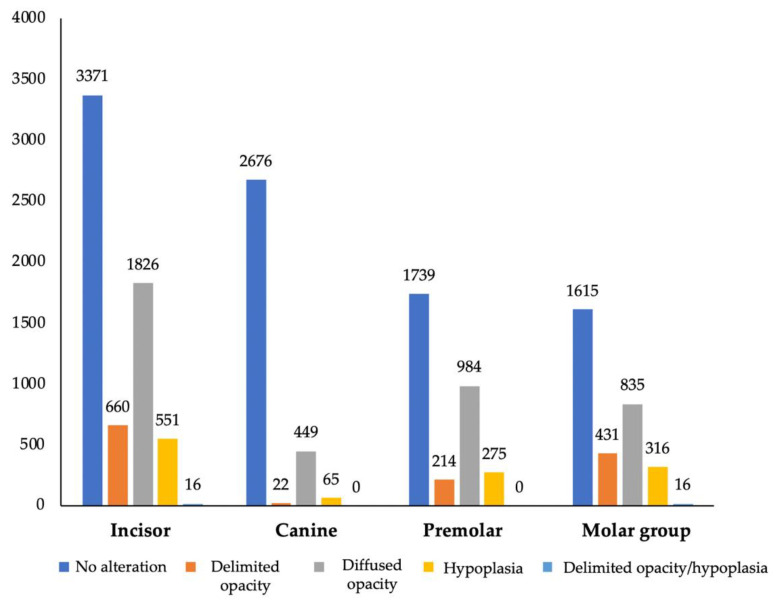
Distribution of types of DDEs by dental group.

Considering all teeth examined, in all age groups, diffuse opacity was more prevalent in all dental groups. Both delimited opacity and hypoplasia were more prevalent in the incisor and molar groups (Figure 1). 

## 4. Discussion

This research represents the first report of DDEs in permanent dentition in Ecuador carried out on schoolchildren aged 6 to 12 years from three provinces in southern Ecuador. The prevalence of all types of DDEs among the 1606 children examined was 50.5%, with a defect present in at least one tooth. These findings are consistent with the results of other studies, especially in low-income populations [28,29]. The increased risk of DDEs in the permanent dentition is probably related to a critical period of amelogenesis between 0 and 2 years of age, when the child is particularly vulnerable to several common systemic conditions that can affect enamel development [1,6,28]. 

The most common type of defect was diffused opacities (26.3%), which is consistent with the findings of previous studies [30,31]. Studies indicate that diffuse opacity is directly related to fluoride consumption [15], being the most common defect found in populations with fluoridated water supply, while in regions without or with low levels of fluoride in drinking water, the most common defect is delimited opacity [32,33]. In the case of the southern region of Ecuador, there are reports of the presence of high levels of fluoride in the water. This chemical element has been associated as a contaminant responsible for pathologies at the level of dental enamel [34] when administered in the stages of dental formation [34,35,36] or due to the involuntary consumption of toothpaste already reported in studies carried out in Ecuador [25,37]. 

The results showed that in the southern region of Ecuador there were no statistically significant differences between the three provinces studied; however, it was observed that the prevalence of hypoplasia was higher in the rural areas. These differences could be explained by the higher rate of children with chronic or acute malnutrition and children with very low birth weight [38]. The presence of hypoplasia invites health authorities to develop and promote health measures that reduce the association of this pathology with the presence of caries and irregularities in dental surfaces [11,23,28,29,35,39,40], which can easily lead to an irreversible loss of dentin [41].

No significant differences were observed in the presence of the other DDEs between urban and rural areas, coinciding with previous studies [4,15,21,40]; however reported findings from Saudi Arabia and Australia [4,15] found a higher prevalence of DDEs in rural areas and areas of extreme poverty, relating to predisposing factors present in rural areas of Ecuador. 

Regarding the groups of teeth most affected and the presence of DDEs detected, the study showed that incisors and molars were the most affected groups, coinciding with what has been reported in other studies, associating their presence with greater exposure to fluoride [40,42], dependent on the time of permanence of the tooth in the mouth and the concentration of the chemical that have been associated with a cumulative effect of fluoride in the tooth [43]. The later a tooth is mineralized, the greater the prevalence and severity of enamel alterations [14]. This situation would explain the low presence of DDEs in the premolars and molars of the participants under 12 years of age. The presence of detected opacities in this work was shown symmetrically on both sides of the arch, differing from the findings reported in previous studies [44], in which the severity of the opacities was evidenced unilaterally, which was associated with the course of blood flow and blood vessels [39], which would trigger a greater presence of DDEs on one side of the arch. The strong esthetic compromise given by the presence of these stains negatively influences the quality of life of those who suffer from them and their close family nucleus [40]. Fluorides have not always been related as the only cause of the appearance of diffuse opacities in the enamel. Certain medications such as amoxicillin administered during early childhood have been related to the appearance of these defects; however, more studies are needed that allow concluding this association [41]. 

Among the limitations of the study, we can indicate that we did not develop a nutritional evaluation for those involved in this research, since nutritional status is possibly closely linked to DDEs. Some studies have already reported this possible association, although this should be analyzed with caution since it will depend on the type of dentition analyzed and the age of the individuals. It must be considered that nutritional status is also linked to socioeconomic level. Children with limited resources and in rural areas are more exposed to nutritional deficiencies and therefore problems at the oral level [15,16].

Another of the limitations of the study was the evident possibility of bias in the detection of the pathologies, which is inevitable, despite the training and standardization of the observer. Elements such as the light that falls on the dental surfaces and the possible presence of biofilm even after brushing the teeth of the participant can often act as distracting elements even in the face of a trained examining eye, which can often become exhausted in this type of study, which invites the search for new strategies for collecting information. 

## 5. Conclusions

The prevalence of developmental enamel defects in children in the southern region of Ecuador is high, with diffuse opacities being the most frequently found type of DDEs, followed by delimited opacities and hypoplasia. The evident presence of enamel alterations in the population evaluated invites us as clinicians to evaluate the risk of developing carious alterations after their presence, associated with the accumulation of bacterial plaque in the irregularities of the surfaces involved with these alterations. 

Although health agencies worldwide have directed their strategies to control carious lesions by incorporating fluoride in the water, the lack of regulation and monitoring of this strategy has often led to triggering qualitative and quantitative pathologies in the enamel, making the structuring of control strategies a challenge, through longitudinal and follow-up studies involving standardized timely diagnostic procedures that limit lesions and their consequences.

## Figures and Tables

**Table 1:** Distribution according to sex and age.

		Without DDEs		With DDEs		X^2^	*p*
		n	%	n	%		
Sex	Male	414	50.8	408	49.4	0.205	0.651
	Female	386	49.2	398	50.6		
	Total	800	100%	806	100%		
Years	Six	20	48.8	21	51.2	4.265	0.641
	Seven	141	55.3	114	44.7		
	Eight	125	47.7	137	52.3		
	Nine	125	48.3	134	51.7		
	Ten	126	50.6	123	49.4		
	Eleven	122	49.6	124	50.4		
	Twelve	141	48.0	153	52.0		

Note: X^2^ = chi-square value; *p* = *p*-value—statistical significance.

**Table 2:** Distribution of types of DDEs by sex and age.

Type of DDEs	Sex		Years						
	Male	Female	Six	Seven	Eight	Nine	Ten	Eleven	Twelve
Delimited opacity	15.90%	12.10%	9.80%	9.40%	15.60%	11.60%	7.60%	15.00%	12.20%
Difused Opacity	25.10%	27.60%	34.10%	28.60%	27.50%	26.30%	24.10%	25.20%	22.60%
Hypoplasia	8.60%	10.70%	7.30%	6.70%	9.50%	12.70%	16.90%	11.00%	26.90%
Delimited opacity/hypoplasia	0.50%	0.50%	0.00%	0.00%	0.40%	1.50%	0.00%	0.80%	0.30%
X^2^ (*p*)	0.689 (0.406)		13.35 (*p* < 0.01 *)						

Note: X^2^ = chi-square value; *p* = *p*-value—statistical significance; * significant difference.

**Table 3:** Distribution of DDE types according to urban/rural environment in the provinces of Cañar, Azuay, and Morona Santiago.

Type of DDEs	Azuay		Cañar		Morona Santiago		Total
	Urban	Rural	Urban	Rural	Urban	Rural	
Normal	49.0%	47.4%	49.3%	46.8%	53.2%	54.3%	49.5%
Delimited Opacity	13.9%	7.0%	16.7%	18.0%	12.3%	13.8%	14.1%
Diffused Opacity	26.7%	31.6%	26.2%	25.2%	26.8%	20.7%	26.3%
Hypoplasia	9.6%	13.2%	7.7%	9.8%	7.2%	10.6%	9.7%
X^2^ (*p*)	0.144 (0.705)		0.363 (0.547)		0.048 (0.827)		

Note: U = urban; R = rural; X^2^ = chi-square value; *p* = *p*-value—statistical significance.

## Data Availability

https://ucacueedu-my.sharepoint.com/personal/mvelezl_ucacue_edu_ec/_layouts/15/onedrive.aspx?ga=1&id=%2Fpersonal%2Fmvelezl%5Fucacue%5Fedu%5Fec%2FDocuments%2FBase%20nueva%20Eleonor%20Velez%20%281%29%2Esav&parent=%2Fpersonal%2Fmvelezl%5Fucacue%5Fedu%5Fec%2FDocuments&p=14 (accessed on 18 December 2021).
